# Editorial: Beta-Cell Fate: From Gene Circuits to Disease Mechanisms

**DOI:** 10.3389/fgene.2022.822440

**Published:** 2022-02-25

**Authors:** Luiza Ghila, Kenichiro Furuyama, Shane T. Grey, Hanne Scholz, Simona Chera

**Affiliations:** ^1^ Center for Diabetes Research, Department of Clinical Science, Faculty of Medicine, University of Bergen, Bergen, Norway; ^2^ Center for iPS Cell Research and Application (CiRA), Kyoto University, Kyoto, Japan; ^3^ Immunology Department, Garvan Institute of Medical Research, Darlinghurst, NSW, Australia; ^4^ Faculty of Medicine, St Vincent’s Clinical School, University of New South Wales Sydney, Sydney, NSW, Australia; ^5^ Hybrid Technology Hub-Centre of Excellence, Faculty of Medicine, University of Oslo, Oslo, Norway; ^6^ Department of Transplant Medicine and Institute for Surgical Research, Oslo University Hospital, Oslo, Norway

**Keywords:** MODY (mature onset diabetes of the young), T1D (type 1 diabetes), T2D (type 2 diabetes), mRNA processing, UPR stress, islet heterogeneity, cell reprogramming

Diabetes represents a group of energy metabolism pathologies where the most common forms of the disease exhibit a polygenic and multifactorial aetiology (American Diabetes Association, 2005). Diabetes has truly reached worldwide epidemic proportions with 537 million people living with diabetes worldwide (International Diabetes Federation, 2021). While diabetes can be managed, for many people the onset of life-threatening complications including blindness, kidney failure, heart attacks, stroke, and lower limb amputation further exacerbates the impact on mortality and morbidity (6.7 million deaths related to diabetes complications were reported in 2021). The major difficulty is distinguishing from the plethora of modulations the ones directly responsible for disease initiation. To date it remains largely unknown what are the external factors and cellular signals leading to insulin-producing β-cells decay or dysfunction are and what molecular mechanisms and cellular processes characterize this transition. Identifying the mechanisms governing the onset and progression of these complex conditions is exceedingly challenging, due to their multifactorial environmental component and the intricate genetic susceptibility interaction. Nevertheless, the past decade registered important advances towards understanding these issues. Moreover, the role of the other pancreatic cell populations in the onset, progression, and treatment of diabetes started to be revealed (Chera and Herrera, 2016). These advances contribute towards a more comprehensive demultiplexing of the large diversity of diabetes mechanisms, which is expected to critically contribute to an unambiguous diabetes reclassification.

The aim of this Research Topic was to provide a snapshot of current studies focused on molecular circuits and cellular processes involved in the development, function, and dysfunction of β-cells, touching multiple aspects of islet niche and disease progression.

## Molecular Level/Novel Molecular Players

Both T1D and T2D have a complex polygenic and multifactorial aetiology, hence providing a highly variable cellular and molecular readout, even between genetically related individuals. Due to this intrinsic high level of complexity, caused by the overlap between environment and intricate genetic susceptibility, there are still not clearly defined molecular and cellular mechanisms of disease onset and progression. Conversely, monogenic disorders are caused by single gene defects occurring in all cells of the organism. Therefore, the characterization of the causative genes and their associated molecular mechanisms involved in monogenic diabetes onset contribute to understanding the complex non-inherited T1D or T2D disorders.

To date, the genetic predisposition for monogenic diabetes was mostly studied in populations from western countries. To bridge this gap, Zhong et al. analyzed the clinical and genetic characteristics of 200 diabetic patients from Northern China to map the monogenic diabetes prevalence and identify putative novel mutations responsible for MODY. The study pinpointed a heterozygous missense mutation in the coding region of FOXM1 (rs535471991) as a potentially pathogenic variant, most likely affecting the risk of MODY. In the same line, Lin et al. described a case of ABCC8-MODY, previously unreported in China.

Furthermore, by analyzing 160 Egyptian patients, Kassem et al. investigated the correlation between two SERPINB1 SNPs and type 2 diabetes risk. They revealed that SERPINB1 SNP rs152826 can potentially predict glycemic control in diabetic patients while suggesting that the AA genotype of this SNP can be associated with an overall better glycemic regulation. In contrast, the G allele might be a “risk allele” for poor glycemic control.

Although numerous human participants were recruited for genome-wide association studies (GWAS), the genome and environmental factors (lifestyle) variability usually confound datasets, especially when studying a complex trait like blood glucose homeostasis. Hence, meticulous animal model studies are still a critical tool in diabetes research allowing accurate manipulation of both environment and genetic variance. In an original research article, Aga, Hallahan et al. crossed normoglycemic lean inbred DBA mouse with the diabetes-prone New Zealand obese (NZO) strain to identify novel potential T2D genes by using positional cloning. Focused on the most prominent diabetes quantitative trait loci (QTL) Nidd/DBA on chromosome 4, this study identified Kti12, Osbpl9, Ttc39a, and Calr4 as potential T2D candidates.

Interestingly, the associated loci for T1D and T2D seem to be almost completely separated, despite a partially shared phenotype. However, some genes from the risk loci for T1D and T2D might actually interact in common networks in order to mutually regulate crucial pancreatic islet functions. In order to investigate this avenue, Kaur et al. used a dual systems genetics approach by analyzing 57 T1D and 243 T2D established GWAS loci. This study identified a number of novel plausible common candidate genes and pathways for T1D and T2D: nine genes in common T1D and T2D loci that harbor islet eQTLs in linkage disequilibrium with disease-associated variants (GSDMB, CARD9, DNLZ, ERAP1, PPIP5K2, TMEM69, SDCCAG3, PLEKHA1, and HEMK1), and four genes in common T1D and T2D loci mutually regulated by palmitate and cytokines (ASCC2, HIBADH, RASGRP1, and SRGAP2).

Prolonged chronic hyperglycemia is a leading risk factor for developing micro- and macrovascular complications. Interestingly, some individuals do not develop these complications, despite long disease duration suggesting the involvement of protective mechanisms in these patients or, alternatively, the presence of risk factors in the patients that do progress to complications. Keindl et al. studied a panel of inflammatory markers in the plasma of long-term T1D patients with and without vascular complications and found that increased plasma level of a cytokine, soluble interleukin-2 receptor alpha (sIL-2R), was positively associated with the presence of vascular complications.

## Cellular Level/Novel Cellular Processes

As also seen above, numerous alleles and mutations associated with increased risk of developing T1D or T2D diabetes (Fuchsberger et al., 2016) were identified in recent years, however cellular fate determination, identity, and function are also regulated at other levels, i.e. mRNA processing or protein folding, packing and sorting. Moss and Sussel summarise in a review the current knowledge on RNA-binding proteins, alternative-splicing events, and transcriptome-wide changes in RNA methylation landscape influencing specific functions of insulin-producing β-cells. As islet cells are mainly secretory cells handling a high amount of hormone production, there is strong pressure on the endoplasmic reticulum (ER) intrinsic folding capacity. This might lead to, for example, misfolded pro-insulin in β-cells triggering a physiological upregulation of the unfolded protein response (UPR) to restore homeostasis. Lenghel et al. discussed recent data addressing the role of UPR on the dedifferentiation or proliferation of β-cells, as well as in triggering inflammation at the islet level, and proposed possible therapies by using UPR for restoring β-cells homeostasis according to their stress level.

## Islet Level/Regenerative Strategies

An increasing amount of recent studies suggest that diabetes is not only a β-cell disease, and several other islet cell types could also contribute to its physiopathology (Chakravarthy et al., 2017; Holst et al., 2017; Traub et al., 2017; Brissova et al., 2018; Cigliola et al., 2018; Rorsman and Huising, 2018; Furuyama et al., 2019; Cigliola et al., 2020; Nair et al., 2020). In a mini-review Bru-Tari et al. discussed recent work on islet cell heterogeneity and how this knowledge can be used to restore islet function and therefore to improve current β-cell replacement therapies. An alternative therapeutic strategy could be aimed at increasing the replication rate of insulin-producing β-cells (Nir et al., 2007), which is at very low levels in homeostatic islets. Along these lines, Jiang et al. studied the role of SNAPIN, a protein that interacts with SNARE complexes, in mediating β-cell proliferation. One potential approved drug with β-cell regenerative potential might be liraglutide, an analogue of glucagon-like peptide-1. Villalba et al. investigated the role of liraglutide on the restoration of β-cell mass in regards to neogenesis and transdifferentiation, although additional lineage-tracing studies will be required to clarify the origin of the cells involved in these processes.

## Perspectives

A major goal of diabetes research is mapping the diverse specific cellular and molecular mechanisms leading to disease onset and progression. Advances in this area will lead to an improved diabetes classification and a more targeted management of the different diabetes subtypes. The need for this fine-tuned reclassification is clearly illustrated by the current high incidence of diabetes-related complications resulting from insufficient disease models supporting appropriate clinical decision-making. Better disease knowledge and, consequently, its improved management will help avoid unnecessary treatments, improve the patients’ quality of living, reduce costs and ultimately bend the mortality curve.

Thus, in our view, demultiplexing the pathophysiological mechanisms characterising diabetes will remain a key goal of diabetes research, which can be achieved by further *1*) mapping associated genetic factors, including the mechanistic characterization and pathogenicity assessment of relevant genetic variants in different ethnic groups; *2*) identifying key regulators controlling the cellular processes and molecular landscape leading to disease onset and complications; 3) developing top-notch cell and animal models for studying islet cell function, dysfunction, and extra-pancreatic confounding effects.
